# Pathogen manipulation of host metabolism: A common strategy for immune evasion

**DOI:** 10.1371/journal.ppat.1006669

**Published:** 2017-12-07

**Authors:** Zachary Freyberg, Eric T. Harvill

**Affiliations:** 1 Department of Psychiatry, University of Pittsburgh, Pittsburgh, Pennsylvania, United States of America; 2 Department of Cell Biology, University of Pittsburgh, Pittsburgh, Pennsylvania, United States of America; 3 Center for Vaccine and Immunology, College of Veterinary Medicine, University of Georgia, Athens, Georgia, United States of America; 4 Department of Infectious Diseases, College of Veterinary Medicine, University of Georgia, Athens, Georgia, United States of America; University of Wisconsin Medical School, UNITED STATES

The immune system is one of the three main consumers of energy in the human body; brain, muscle, and the immune system use similar amounts (approximately 500 kcal/day) [1]. Rapid bursts of cellular proliferative, biosynthetic, and secretory activities by leukocytes require considerable metabolic resources that are especially important during periods of infection and inflammation [2, 3]. Because immune cells have negligible intracellular nutrient stores and rely on aerobic glycolysis for activation and proliferation, they are particularly dependent on the uptake of metabolic substrates [2–6]. Indeed, glucose uptake is the primary limiting factor in T-cell activation [5, 6]. T-cell activation and proliferation are decreased in low-glucose states along with reduced production of cytokine effectors of the immune response, including interferon γ (IFN-γ) [6–8]. Susceptibility to infections by individuals with metabolic diseases further underscores the significant impact of metabolic disruption on the functions of innate and adaptive immunity [9, 10].

Pathogens that are well adapted to their hosts have developed an extraordinarily wide range of mechanisms to modulate host immunity in order to facilitate and prolong infection and transmission. Several of these mechanisms are relatively well characterized and appear to act directly on host target tissues. Traditionally, the effects that many pathogens have on host metabolism have been assumed to be downstream consequences of pathogenesis. However, increasing evidence suggests that these pathogen-induced metabolic disturbances may instead reflect aspects of the pathogens’ modulation of the immune response to enhance and/or prolong the period of infection and transmissibility (Fig 1). Here, we examine three diverse but highly prevalent global pathogens that disrupt host metabolism during infection and may thereby alter the host immune response: *Trypanosoma cruzi*, *Plasmodium falciparum*, and *Bordetella pertussis*, responsible for Chagas disease, malaria, and whooping cough, respectively. Considering how metabolic changes that pathogens induce in the host can affect the immune response may reveal commonalities that can contribute to understanding, controlling, and treating a wide range of diseases.

**Fig 1:**
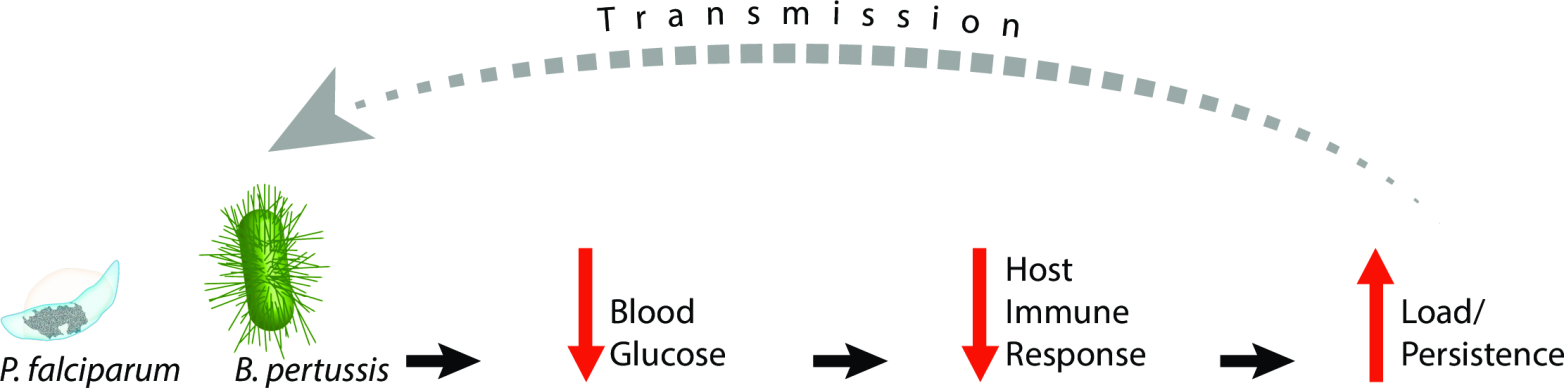
Model of pathogen interactions between host metabolism and immune response. We propose a generalizable model demonstrating the means by which pathogens manipulate host metabolism to facilitate infection. Diverse pathogens, including *P*. *falciparum* and *B*. *pertussis*, decrease host blood glucose levels. This results in an impaired host immune response that consequently exacerbates and/or prolongs infection by providing the pathogen with improved opportunities to increase load and/or persistence and enhance transmission to other hosts.

## T. cruzi

*T*. *cruzi* disrupts host glucose homeostasis within 30 days post infection (DPI) [11, 12]. Parasites are detected as early as 15 DPI within insulin-secreting pancreatic beta cells. *T*. *cruzi*’s actions in the pancreas are quite selective. Pancreatic islet architecture is significantly disrupted such that beta cells are preferentially targeted by the parasite, and other islet cell types such as glucagon-secreting alpha cells are spared [12].

Acute and subacute *T*. *cruzi* infection produce a complex pattern of changes in host insulin secretion characterized by hypoinsulinemia with accompanying hyperglycemia [13]. During acute infection, *T*. *cruzi* impairs hepatic gluconeogenesis and induces a strong inflammatory response within the host capable of triggering a systemic “cytokine storm.” This buildup of cytokines leads to decreased feeding by the host and increased glucose uptake by the parasites [14]. In chronic infection, there is impaired insulin secretion from pancreatic beta cells secondary to the ability of *T*. *cruzi* to modify insulin granule fusion, resulting in failure to properly release insulin rather than a defect in hormone production [11, 12]. Additionally, other contributors to hypoinsulinemia during infection include pathogen-induced autonomic disruption of the parasympathetic innervation of the pancreas through denervation [15, 16]. Because pancreatic secretion of insulin relies on parasympathetic neuronal inputs [17], parasympathetic denervation may contribute to the diminished insulin secretion in Chagas disease [13]. Infection also elevates glucagon levels, which further disrupts glucose homeostasis and leads to host hyperglycemia [13]. However, given that most of the above studies were conducted in animal models, it remains unclear whether *T*. *cruzi*-induced metabolic phenomena are also evident in humans. Clinical studies demonstrating both hyperglycemia and hypoinsulinemia in patients with Chagas disease have been inconclusive or variable [18]. Nevertheless, an examination of subgroups of Chagas patients reveals significant abnormalities in glucose metabolism, including hypoinsulinemia, hyperglycemia, and glucose intolerance [18–20]. This suggests that some patients may be especially vulnerable to the metabolic consequences of *T*. *cruzi* infection. Future work is needed to clarify the factors responsible for such selective clinical vulnerability to metabolic disruption in Chagas patients and the specific mechanisms employed by *T*. *cruzi* to target these affected patients.

## P. falciparum

*P*. *falciparum* induces hyperinsulinemia and hypoglycemia during infection [21–24]. These metabolic sequelae are associated with more severe morbidity and increased mortality in malaria [25, 26]. However, the mechanisms for malaria-induced hypoglycemia remain poorly understood.

Diminished hepatic gluconeogenesis coupled with increased metabolic demands arising from infection have been proposed as important contributors to host hypoglycemia [27, 28]. Intriguingly, *P*. *falciparum* appears to act directly on pancreatic beta cells to cause insulin hypersecretion and, ultimately, hypoglycemia. Treatment of cultured pancreatic beta cells with plasma from patients with malaria-induced hypoglycemia resulted in a significant increase in insulin secretion [21]. Significantly, these metabolic effects were attenuated in diabetic animals whose pancreatic beta cells were depleted via the beta cell toxin streptozotocin [23, 24], further implicating *P*. *falciparum*’s effects on these beta cells in metabolic disruption. Overall, these studies suggest that *P*. *falciparum* secretes factors and activates the host immune system to increase pancreatic beta cell insulin secretion, which contributes to hypoglycemia.

## B. pertussis

*B*. *pertussis*’ expression of virulence factors plays a large role in pertussis illness. While these factors promote bacterial adhesion and invasion locally, they also act more globally as immunomodulators that subvert host innate and adaptive immunity [29–31]. Consequently, even though *B*. *pertussis* is best known for its actions on the respiratory tract, this pathogen also acts at several other host sites, including spleen and blood; both sites play direct roles in mobilizing the host immune response during different phases of infection [31]. Significantly, *B*. *pertussis* also has a profound effect on host metabolism.

As early as the 1930s, clinical reports described hyperinsulinemia and resultant long-lasting hypoglycemic states during *B*. *pertussis* infection [32]. Furthermore, *B*. *pertussis*-induced hyperinsulinemia and hypoglycemia were shown to significantly increase susceptibility to inflammatory and anaphylactoid reactions [33, 34]. Beginning in the 1960s, mouse models recapitulated *B*. *pertussis*-induced hypoglycemia and hyperinsulinemia [32, 35, 36]. Moreover, in vivo studies demonstrated that selective destruction of beta cells using the drug alloxan attenuated this *B*. *pertussis*-induced hyperinsulinemia and hypoglycemia [32]. These data thus suggest that, as with *P*. *falciparum*, beta cells within the pancreatic islet are specifically targeted to stimulate hyperinsulinemia during infection.

It was discovered that these metabolic effects were primarily caused by a virulence factor secreted by *B*. *pertussis* originally named islet-activating protein (IAP). Though subsequently renamed pertussis toxin (PTX), this toxin was initially isolated and studied based on its direct actions on pancreatic beta cells to stimulate insulin secretion [37, 38]. PTX was later found in the circulating serum of *B*. *pertussis*-infected animals, providing a route for its systemic actions [39].

A crucial clue in elucidating the mechanisms by which *B*. *pertussis* and other pathogens exert metabolic effects within the host may lie in the actions of secreted virulence factors such as PTX and adenylate cyclase toxin (ACT). Secretion of these factors is part of a common strategy used by pathogens to facilitate infection and pathogenesis. *Mycobacterium tuberculosis*, *Bacillus anthracis*, *Salmonella enterica*, and *Listeria monocytogenes* rely on multiple, complex secretion mechanisms for adhesion, evasion of host defenses, and virulence, among a plethora of other functions [40, 41], but the potential impact on metabolism is somewhat more complex and has been less well considered. Understanding how *B*. *pertussis*-secreted factors disrupt metabolism to affect pathogenesis and immunity may, therefore, shed light more generally on this common virulence strategy and provide an example relevant to other diseases.

### PTX

**Fig 2:**
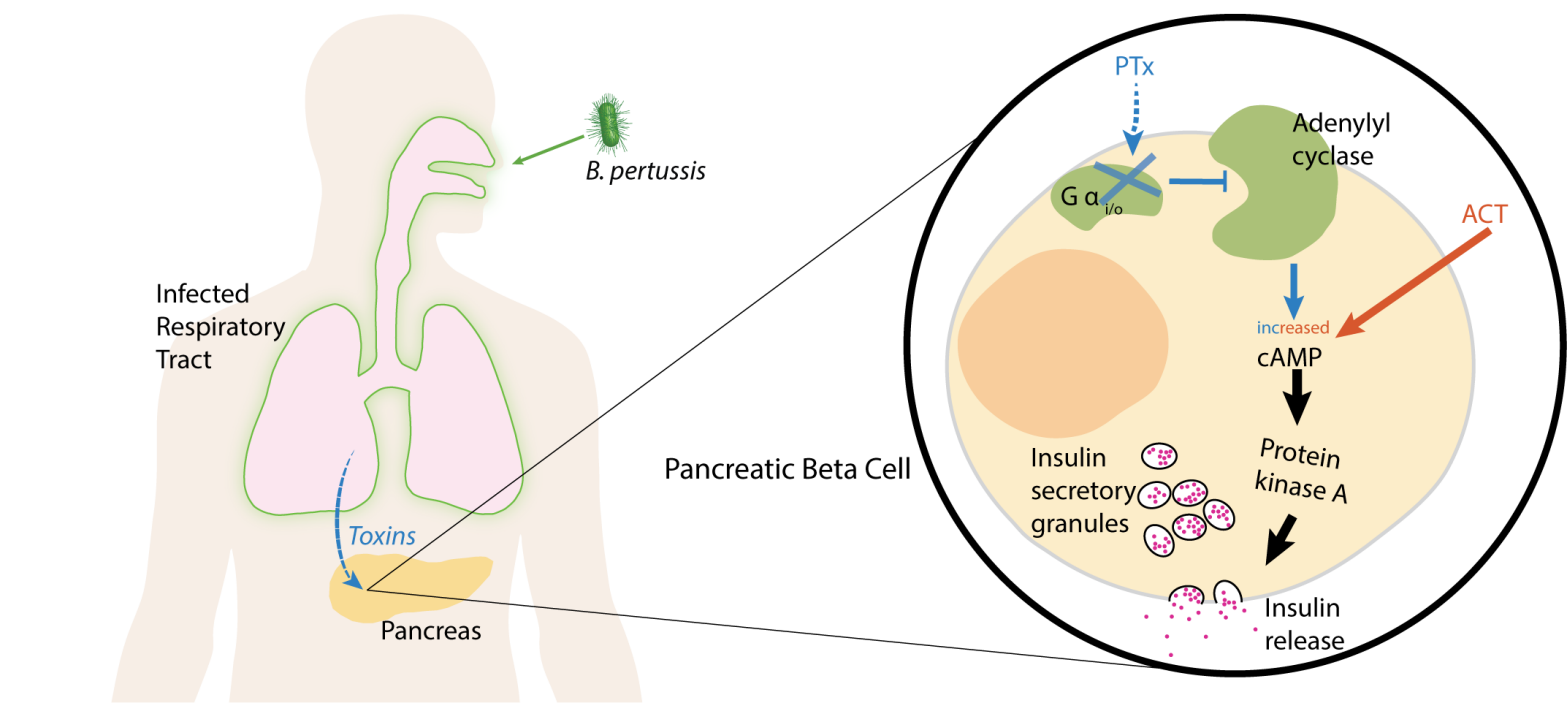
Model for PTX- and ACT-induced hyperinsulinemia. Following infection, *B*. *pertussis* produces PTX, which acts not only within the respiratory tract but also directly on insulin-secreting pancreatic beta cells. Within these cells, PTX inhibits Gα_i/o_ signaling that ordinarily inhibits adenylate cyclase, the enzyme responsible for cAMP synthesis. This leads to increases in cAMP and activates PKA, a key stimulator of insulin release. In parallel, *B*. *pertussis* also secretes ACT, which directly increases cAMP levels to also produce hyperinsulinemia and subsequent hypoglycemia in the host. ACT, adenylate cyclase toxin; PKA, protein kinase A; PTX, pertussis toxin.

PTX is one of the most important virulence factors associated with *B*. *pertussis* pathogenesis [42]. PTX targets G-protein–coupled receptors (GPCRs) and inactivates alpha subunits of the heterotrimeric Gα_i/o_ (Gi/o) protein family immediately downstream of these receptors by adenosine diphosphate (ADP) ribosylation [37]. GPCRs expressed in pancreatic beta cells, including α_2_ adrenergic and dopamine D_2_ receptors, play important roles in modulating insulin secretion. Because these GPCRs are Gi/o coupled, they are directly susceptible to PTX action. Under normal circumstances, GPCR stimulation results in an autocrine negative feedback circuit in which subsequent insulin release is diminished [43, 44]. The mechanism for this negative feedback is based on Gi/o’s ability to reduce levels of a key mediator of insulin secretion, cyclic AMP (cAMP). Gi/o inhibits adenylate cyclase, the enzyme responsible for cAMP synthesis, which thus prevents activation of the cAMP-dependent protein kinase A (PKA), a powerful stimulator of insulin secretion (Fig 2) [43]. This PTX-sensitive, Gi/o-mediated signaling mechanism prevents oversecretion of insulin during glucose stimulation and is important for maintaining metabolic homeostasis [44, 45]. Thus, PTX’s inhibitory actions on GPCR and Gi/o signaling provide a mechanism for the toxin’s stimulation of insulin release [37].

In addition to stimulating insulin secretion, PTX acts on other aspects of host metabolism, including glucose transport. Acute PTX treatment diminishes insulin-stimulated glucose transport activity in key tissue targets of insulin action—myocytes and adipocytes—via the inhibition of Gi/o-mediated signaling independently of its effects on cAMP biosynthesis [46–48]. PTX also reduces insulin receptor affinity to insulin, further potentiating the toxin’s inhibitory effects on insulin-stimulated glucose transport [49]. Moreover, prolonged PTX action produces hypoglycemia by changing glucose transporter (GLUT) expression [50]. PTX increases the expression of GLUT-4 in muscle, resulting in increased transport of blood glucose into muscle, and lowers overall circulating glucose [50].

### ACT action

In concert with PTX secretion, *B*. *pertussis* produces the toxin ACT, which is responsible for several aspects of *B*. *pertussis*’s virulence, including the impairment of T-cell activation and chemotaxis to undermine the host adaptive immune response [51, 52]. ACT is a bifunctional protein composed of an amino terminal adenylate cyclase domain and a repeat in toxin (RTX) domain that forms pores in membranes to facilitate the toxin’s entry into host cells [51]. Notably, the adenylate cyclase domain of ACT bypasses endogenous host adenylate cyclase by exhibiting its own very high catalytic activity [51]. This renders the toxin capable of rapidly generating superphysiological increases in intracellular cAMP levels within seconds of entry into host cells [53]. Therefore, analogous to PTX action in pancreatic beta cells, it is possible that ACT also acts on beta cells to increase cAMP, activate PKA, and thus significantly elevate insulin secretion.

## Effects of host immune response on metabolism during infection

In addition to direct actions of pathogen-secreted toxins on host tissues to induce hypoglycemia, pathogens may also manipulate the immune response to disrupt host metabolism. For example, infection can trigger host immune cell production of cytokines, including interleukin-1 (IL-1). IL-1 activates the sympathetic nervous system and causes a drop in blood glucose [54]. Other cytokines, including IL-10, have been implicated in *P*. *falciparum*-induced hypoglycemia [55]. Likewise, *Plasmodium* spp. elicit the production of host immunomodulators such as tumor necrosis factor α (TNFα) during infection [56, 57], which also produces hypoglycemia [58]. Consequently, given the significant correlation between TNFα levels and hypoglycemia in severe malaria and cerebral malaria, it has been suggested that TNFα be used as a potential prognostic indicator for disease severity [59]. Given TNFα’s role as a proinflammatory cytokine, this hypoglycemia may result either through direct or indirect cytokine actions on host metabolic and immune functions or, more likely, some combination of both [60]. Future work is clearly needed to disentangle the mechanisms of TNFα’s metabolic effects during infection.

Like *P*. *falciparum*, *B*. *pertussis* infection increases host IL-1 production as well as raises levels of IL-17, which is also associated with hypoglycemia [30, 61]. Moreover, ACT action has been implicated in boosting host IL-10 production; IL-10 not only fosters hypoglycemia but also impedes the development of host adaptive immunity [62]. Unlike *P*. *falciparum*, however, *B*. *pertussis* toxins PTX and ACT inhibit TNFα production through their stimulation of cAMP synthesis in monocyte-derived dendritic cells [63].

## Clinical implications

Despite numerous differences between *B*. *pertussis* and *P*. *falciparum*, they share the ability to cause profound disturbances in host metabolism during infection. Because hypoglycemia is often associated as a marker for disease severity, we propose that *P*. *falciparum* and *B*. *pertussis* induction of hypoglycemia serve as model systems to answer fundamental questions concerning how pathogen manipulation of metabolism can affect infection, pathogenesis, and the host immune response.

These experimental systems can address several important questions relevant to these and other diseases, such as the following:

1. Do pathogen-induced hyperinsulinemia and hypoglycemia modify the host immune response to enhance and/or prolong pathogenesis and infection?
2. Does correction of hypoglycemia during infection impact pathogen growth, persistence, and pathogenesis?
3. Do secreted toxins such as ACT and PTX act directly on insulin-secreting pancreatic beta cells to cause hyperinsulinemia? If so, what are the individual and collective contributions of the toxins and G-protein–mediated signaling to these metabolic disruptions?

Host immune cells depend on circulating blood glucose to supply adequate substrates for their metabolic requirements, particularly for their rapid activation and expansion during infection. Therefore, we propose that pathogen-induced hypoglycemia deprives the immune system of the energy required to mount an effective inflammatory and/or adaptive immune response. In contrast, unlike host lymphocytes, *B*. *pertussis* is largely resistant to the hypoglycemic state it induces because it is incapable of utilizing glucose as its main carbon source for generating energy owing to an incomplete citric acid cycle [64]. On the other hand, *P*. *falciparum* relies on glycolysis and the tricarboxylic acid cycle, making it likely more sensitive to changes in host blood glucose levels than *B*. *pertussis* [65].

An important implication of our reasoning is the prediction that correcting hypoglycemia will limit the duration and/or severity of infection. Such an approach may, therefore, constitute a new avenue of therapeutic interventions for the metabolic sequelae of infection. To date, therapeutic interventions to correct pathogen-induced metabolic disturbances have not been directly tested clinically. Nevertheless, there is encouraging evidence in animal models of diabetes that diabetic hyperglycemia can moderate sequelae of *P*. *falciparum* infection. Parasitemia during malaria was significantly lower in moderately diabetic animals compared with normal mice [23]. These findings suggested that raising blood glucose to counteract *P*. *falciparum*’s attempts to induce host hypoglycemia may limit pathogenesis. Indeed, some have argued that metabolic diseases such as diabetes may confer a selective advantage in some populations, in part because altered metabolic signaling pathways that are targeted by pathogens may protect the host from aspects of infectious disease [23, 66]. In contrast, in the setting of diabetic hyperglycemia, *T*. *cruzi* infection parasitemia and mortality were significantly increased [67], suggesting that, for this pathogen, diabetic hyperglycemia further erodes the capacity of the immune system to control infection. Taken together, these data support both the concept that the disruption of glucose homeostasis by pathogens impairs the host’s capability to effectively control infection and the prospect of simple clinical interventions that can modulate these effects.

## Conclusions and future directions

The manipulation of the host’s metabolic state may not only affect the immune response to the respective causative pathogen but also to additional opportunistic infections, further compounding morbidity and mortality associated with infection. On the other hand, treating the metabolic manifestations of infection, including hyperinsulinemia and hypoglycemia, may potentially blunt or ameliorate the disease course of these pathogens and could be implemented relatively quickly, safely, and inexpensively to make a difference in the lives of many affected people globally.
